# CdSe/ZnS Quantum Dots-Labeled Mesenchymal Stem Cells for Targeted Fluorescence Imaging of Pancreas Tissues and Therapy of Type 1 Diabetic Rats

**DOI:** 10.1186/s11671-015-0959-3

**Published:** 2015-06-13

**Authors:** Haoqi Liu, Wei Tang, Chao Li, Pinlei Lv, Zheng Wang, Yanlei Liu, Cunlei Zhang, Yi Bao, Haiyan Chen, Xiangying Meng, Yan Song, Xiaoling Xia, Fei Pan, Daxiang Cui, Yongquan Shi

**Affiliations:** Department of Endocrinology and Metabolism, Changzheng Hospital, Second Military Medical University, 415 Fengyang Road, Shanghai, 200003 People’s Republic of China; Institute of Nano Biomedicine and Engineering, Key Laboratory for Thin Film and Microfabrication of Ministry of Education, Department of Instrument Science and Engineering, School of Electronics Information and Electronical Engineering, Collaborative Innovational Center for System Biology, National Center for Translational Medicine, Shanghai Jiao Tong University, 800Dongchuan Road, Shanghai, 200240 People’s Republic of China; Department of Digestion, Renji Hospital, School of Medicine, Shanghai Jiao Tong University, Shanghai, 200001 People’s Republic of China; Department of Endocrinology and Metabolism, Dahua Hospital, 901Laohumin Road, Shanghai, 200031 People’s Republic of China

**Keywords:** Mesenchymal stem cell, CdSe/ZnS quantum dots, Fluorescence imaging, Cell labeling, Type 1 diabetes mellitus, Rat model

## Abstract

Mesenchymal stem cells (MSCs) have been used for therapy of type 1 diabetes mellitus. However, the in vivo distribution and therapeutic effects of transplanted MSCs are not clarified well. Herein, we reported that CdSe/ZnS quantum dots-labeled MSCs were prepared for targeted fluorescence imaging and therapy of pancreas tissues in rat models with type 1 diabetes. CdSe/ZnS quantum dots were synthesized, their biocompatibility was evaluated, and then, the appropriate concentration of quantum dots was selected to label MSCs. CdSe/ZnS quantum dots-labeled MSCs were injected into mouse models with type 1 diabetes via tail vessel and then were observed by using the Bruker In-Vivo F PRO system, and the blood glucose levels were monitored for 8 weeks. Results showed that prepared CdSe/ZnS quantum dots owned good biocompatibility. Significant differences existed in distribution of quantum dots-labeled MSCs between normal control rats and diabetic rats (*p* < 0.05). The ratios of the fluorescence intensity (RFI) analysis showed an accumulation rate of MSCs in the pancreas of rats in the diabetes group, and was about 32 %, while that in the normal control group rats was about 18 %. The blood glucose levels were also monitored for 8 weeks after quantum dots-labeled MSC injection. Statistical differences existed between the blood glucose levels of the diabetic rat control group and MSC-injected diabetic rat group (*p* < 0.01), and the MSC-injected diabetic rat group displayed lower blood glucose levels. In conclusion, CdSe/ZnS-labeled MSCs can target in vivo pancreas tissues in diabetic rats, and significantly reduce the blood glucose levels in diabetic rats, and own potential application in therapy of diabetic patients in the near future.

## Background

The amount of diabetic patients has been increasing rapidly. According to the latest report, in China, the proportion of diabetic patients has reached to 11.6 %, accounting for one third of global diabetic patients. How to prevent and treat diabetes has become a challengeable problem. Type 1 diabetes mellitus is characterized by the permanent destruction of pancreatic β cells. Symptomatic individuals typically have lost more than 80 % of β cell population, resulting in essentially no insulin production and an inability to regulate plasma glucose levels properly [1].

Up to date, regarding the therapy of type 1 diabetes, some methods have been reported. For example, cell-based treatments with the aim of recovering pancreatic β cell function, including the pancreas transplantation, islet transplantation, and stem cell transplantation, focusing on replacing damaged β cell populations, should be the ideal potential therapeutic pathways [2]. Among the cell-based treatments, the current gold standard is whole-pancreas transplantation. Recent pancreas transplants have demonstrated sustained exogenous insulin independence over 2 years post-transplant, evidenced by normal HbA1c values. However, pancreas transplantation is a major surgical procedure with mortality rates ranging from 1 to 3 %, and the complications of the procedure required long-term immunosuppression [3]. In order to overcome these shortcomings of whole-organ transplantation, pancreatic islet cells transplanting has been actively explored. In 2000, a corticosteroid-free immunosuppressive regimen, the novel Edmonton protocol, enabled seven patients to remain insulin-independent for an average of 11.9 months [4]. However, these results are quite difficult to reproduce, and nine-year islet graft survival rates are below 10 %. Therefore, looking for new therapeutic method may be a good choice.

Stem cell transplantation for diabetes therapy may offer a promising possibility that deserves to be explored. Stem cell transplantation has some advantages, such as the easy operation, short operation time, and low invasiveness for patients relatively compared with whole-organ transplantation [5]. Up to date, mesenchymal stem cells (MSCs) have been actively investigated for their therapeutic values on different kinds of diseases such as diabetes, injury, gastric cancer, etc. The benefit of MSCs, including multi-lineage differentiation and immunosuppressive capability, suggests a role of MSC therapy for tissue regeneration [6]. However, the distribution and therapeutic effects of transplanted MSCs in diabetic patients are not clarified well.

In recent years, nanotechnology has become an emerging field of interest. Interaction studies between nanomaterials and stem cells have made great advances. The importance of nanotechnology to the fundamental developments in stem cell-based therapies for injuries and degenerative diseases has been recognized. In particular, the effects of the structure and properties of nanomaterials on the tracing of stem cells have become a new interdisciplinary frontier in regeneration medicine and material science.

Quantum dots (QDs) are one kind of nanomaterial which have broad application prospects in cellular imaging, immunoassays, DNA hybridization, and optical barcoding, due to its significant advantages including good photostability, strong fluorescence intensity, and various emission wavelengths. As QDs were broadly used for molecular imaging, QDs biocompatibility attracts more and more attention from the scientific field. Exploration of quantum dots biosafety has become a hotspot. In another aspect, QDs with near-infrared region (NIR) emission have also received increasing attention as an alternative method for overcoming these shortcomings [7, 8].

In our previous study, we have synthesized CdSe/ZnS QDs with near-infrared region [9]. Herein, we evaluated the biosafety of CdSe/ZnS QDs and investigated the in vivo biodistribution and the effects of CdSe/ZnS QDs-labeled MSCs on plasma glucose levels in type 1 diabetes rat models. Our study lays foundation for MSC clinical application for diabetes therapy in the near future.

## Methods

All animal experiments were approved by the Institutional Animal Care and Use Committee of Shanghai Jiao Tong University (NO. SYXK2007-0025).

### Synthesis and Characterization of CdSe/ZnS QDs

CdSe/ZnS QDs were synthesized according to our previous report [9]. Briefly, with the nitrogen protection, 79-mg selenium powder was dissolved in 50-ml liquid paraffin in a three-neck flask and the temperature was increased to 200 °C gradually. After vigorous stirring for 1 h, the mixture was cooled to 80 °C. In another flask, 1.28-g CdO and 11.4-g stearic acid were dissolved in 10-ml liquid paraffin at 160 °C with stirring and protection of nitrogen. When the mixture turned to bright yellow, the cooled solution of Se precursors was rapidly injected into the hot flask containing Cd precursors and the mixture temperature was quickly increased to 200 °C for 90 min. The molar ratio of CdO/Se/stearic acid in liquid paraffin was 1:1:4, and the crude QD products were purified by chloroform and ethanol. In order to improve the optical properties of QDs, an additional semiconductor shell (zinc sulfide (ZnS)) should be coated on CdSe nanocrystals. For the ZnS shell, equal molar ratios of (TMS)_2_S and ZnEt_2_ as precursors of Zn and S and TOP/TOPO were used, and 90 °C was used for shell growth. The final core–shell product was repurified and redispersed into aliquot chloroform for later use. About 10 ml of deionized water was added to the solution to prevent evaporation of chloroform for long-period storage.

### Primary Culture and Identification of Rat MSCs

MSCs were isolated according to our previous reports and were cultured with Dulbecco’s modified Eagle’s medium (DMEM; Gibco, Shanghai, China) with 10 % fetal bovine serum (FBS; Hyclone, Thermo Scientific, Logan, UT, USA), 100 U/ml penicillin, and 100 mg/ml streptomycin (Gibco) at 37 °C in a humidified 5 % CO_2_ incubator. The MSC medium was changed once every 2 days. The primary cells were cultured for 4 ~ 5 days until they reached confluence and were defined as passage“0”. All experiments were performed with the MSCs from passage 3–6. In order to identify MSCs, passage 4 MSCs were detached by 0.25 % trypsin/EDTA and washed twice with phosphate buffered saline (PBS). About 2 × 10^5^ cells were incubated with an appropriate concentration of FITC-conjugated CD29 monoclonal antibody (BioLegend, San Diego, CA, USA), phycoerythrin (PE)-conjugated CD31 monoclonal antibody (BioLegend), APC-conjugated CD45 (BioLegend), and PE-conjugated CD90 (BioLegend) monoclonal antibody for 40 min at 4 °C in a dark environment. MSCs were washed with PBS buffer, were centrifuged for 5 min, and then resuspended in PBS. Quantitative fluorescence analysis was carried out using FACSCalibur cytometer (Becton Dickinson, San Diego, CA, USA) and CellQuest software.

In order to demonstrate the multipotential property of our prepared MSCs, MSCs were further characterized by differentiation assays. Passage 3 MSCs were cultured in an adipogenic medium including DMEM, 5 % FBS, 1-μM dexamethasone (Sigma), 50-μM indomethacin (Sigma), 500-nM IBMX (Sigma), 5 μg/ml insulin (Sigma), 100 U/ml penicillin, and 100 mg/ml streptomycin for about 2 weeks [9]. The medium was replaced every 3 days with a fresh induction medium. Subsequently, to evaluate cultures for adipogenic differentiation, the cells were washed with PBS, fixated with 2.5 % glutaraldehyde, and stained with Oil Red O (Sigma) for revealing lipid globules. In addition, passage 3 MSCs were cultured in DMEM supplemented with 5 % FBS, 50-μM ascorbic acid (Sigma), 10-nM dexamethasone, 100 U/ml penicillin, and 100 mg/ml streptomycin for about 2 weeks for osteogenic differentiation. Finally, the cells were evaluated and cultured for osteogenic differentiation with Alizarin Red S (Sigma).

### Effects of CdSe/ZnS QDs on the Differentiation Ability of MSCs

In order to estimate the changes of MSCs’ differential ability under the condition of CdSe/ZnS, the differentiation experiments were carried out with or without the CdSe/ZnS QDs (16 μg/ml), and the morphology of the final differentiation cells were captured in microscopy and compared.

### Cell Viability Assay

Toxicity test is determined by the cell viability after incubation in a culture medium containing different concentrations of CdSe/ZnS QDs. A controlled trial was conducted by culturing cells in a normal medium without QDs. We carried out this test by using Cell Counting Kit-8 (CCK-8) which is an upgrade alternative to MTT assay. This toxicity assay was performed in a 96-well plate with about 1 × 10^3^ cells seeding in every well. After incubating in a culture medium containing different concentrations (concentration = the volume of QDs/the volume of medium, the unit is μl/ml)of QDs for 24 h, the CCK-8 reagent (10 μl) was added to each well and there, action was allowed to proceed for up to 4 h. The optical absorbance was measured at 450 nm by using the Thermo multiskan MK3 ELISA plate reader according to the protocol of the CCK-8 assay kit.

### Transduction of Different Concentrations of QDs into MSCs

MSCs were cultured in the medium without FBS at 37 °C, in 5 % CO_2_ for 4 h, and different amounts of of QDs(100μg/ml) (5.0, 10.0, 15, and 20 μl/ml) were mixed in the transduction medium (DMEM, 10 % FBS, 100 U/ml penicillin/streptomycin) at 37 °C for 15 min, respectively; the medium was added into the MSC culture flask, incubated in a humidified 5 % CO_2_ incubator at 37 °C for 6 h. And then, the flask was washed three times with PBS and covered with new PBS. The cells were counted and prepared for quantum dot distribution test and cell transplantation.

### The Distribution of the Quantum Dots in MSCs

Passage 3 MSCs were treated with a medium containing CdSe/ZnS QDs (15 μl/ml) for 4 h. Afterward, the cells were visualized under an inverted fluorescence microscope (Olympus IX71, Olympus, Shanghai, China). After incubation with QDs, the cells were fixed with paraformaldehyde for 15 min at room temperature. The labeled MSCs were also stained with 4′,6-diamidino-2-phenylindole (DAPI) in PBS (pH 7.4) for 5 min. And then, the cells were washed with PBS. The cells were observed with the fluorescence microscope. Fluorescence imaging microscopy was equipped with 372- and 510-nm wavelength excitation light filters.

### Preparation of Diabetes Rat Models

Type 1 diabetes SD rat models were prepared according to the previous report [4, 10]. Thirty specimens of eight-week-old male rats were lightly anesthetized. The rats were randomly divided into three groups: the normal control group (*n* = 10), diabetic control group (*n* = 10), and MSC treatment group (*n* = 10). Streptozocin (STZ, Sigma–Aldrich S1030) was dissolved in 0.1-M citrate at pH 4.5 and was immediately injected into the abdominal cavities of the diabetic control group and MSC treatment group at a dose of 60 mg/kg. The normal control group received the same dose of citrate buffer injection. One week later, fast plasma glucose (FPG) amounts of all rats were examined, and those rats with plasma glucose concentration FPG ≥ 16.5 mmol/l were considered as diabetic rats.

### CdSe/ZnS QDs-Labeled MSCs for Targeted Fluorescence Imaging

At the seventh day after the diabetes rat models were prepared, the MSCs (5.0 × 10^6^ cells/rat) labeled with QDs were injected into the rat models in the MSC treatment group via tail vein, while the normal control group rats received the same dose of QDs-labeled MSCs.

For in vivo imaging analysis, whole-animal imaging was performed with the Bruker In-Vivo F PRO system. The images of several time points (1, 3, 6, and 12 h) post-injection of MSCs were captured and analyzed to understand the migration and homing behavior of MSCs in type 1 diabetes rats. The excitation and emission filters were set to 410 and 700 nm (bandpass, ±15 nm), respectively. The collected images were analyzed with the image J software (NIH ImageJ; http://rsb.info.nih.gov/ij/), which employs spectral algorithms to separate autofluorescence from quantum dot signals. (The imaging condition is the same of in vivo imaging.)

### Distribution of CdSe/ZnS QDs-Labeled MSCs in Rats with Type 1 Diabetes

For the ex vivo fluorescence imaging analysis, rats were sacrificed by cervical detachment method at 6 h after MSC injection (time point selection were accorded with the homing effects of MSCs, data not shown), five organs (heart, lungs, pancreas, spleen, and liver) of the normal control and MSC treatment groups were harvested, and the fluorescence images were captured by the Bruker In-Vivo F PRO system. The excitation and emission filters were set to 510 and 700 nm (bandpass, ±15 nm), respectively. The collected images were analyzed with the image J software.

### The Measurement of Plasma Glucose Levels in Diabetes Rat Models

Eight-week-old rats were administered with STZ, which induced pancreatic injury and hyperglycemia. In order to investigate the therapeutic effects of MSCs (unlabeled with QDs), the level of blood glucoses from the 1st to the 8th week after MSC injection were measured by general methods (10 rats of each group, including the normal control, diabetes control, and treatment groups).

### Statistical Analysis

Numerical values are presented as the mean ± SD. Each experiment was repeated three times. Statistical significance was evaluated using unpaired Student’s *t* test for comparisons between the two groups; *p* values <0.05 were considered to be statistically significant. All statistical analyses were performed by the SPSS software package.

## Results and Discussion

### Preparation and Identification of MSCs

**Fig. 1:**
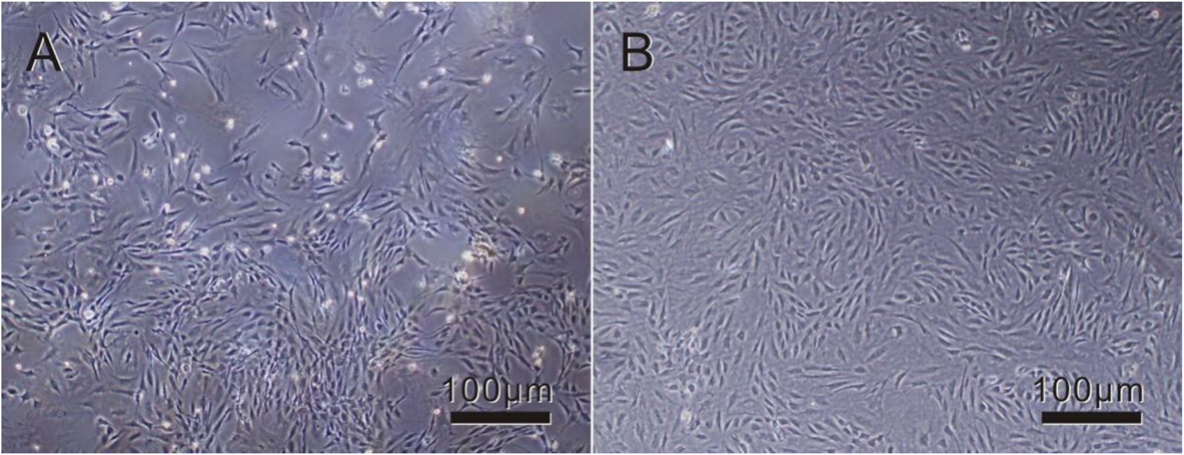
Phase-contrast photo micrographs of cultures showing MSC lines at various passage numbers. **a** During the onset of growth (P0-7th day), MSCs showed diverse morphologies including ovoid, bipolar, and large, flattened morphology. **b** In later passages (P4-4th day), most of these MSCs exhibited large, flattened, or fibroblast-like morphology

Rat MSCs were isolated and cultured. MSCs attached to the culture flasks sparsely and displayed a fibroblast-like, spindle-shaped morphology during the initial days of incubation. After 3 ~ 4 days of incubation, proliferation started and the cells gradually grew into small colonies. One week later, colonies with different sizes increased in number. As growth continued, adjacent colonies interconnected with each other and a monolayer confluence was obtained after 12 ~ 15 days of incubation. In later passages, MSCs exhibited large, flattened, or fibroblast-like morphology (Fig. 1a, b).

**Fig. 2:**
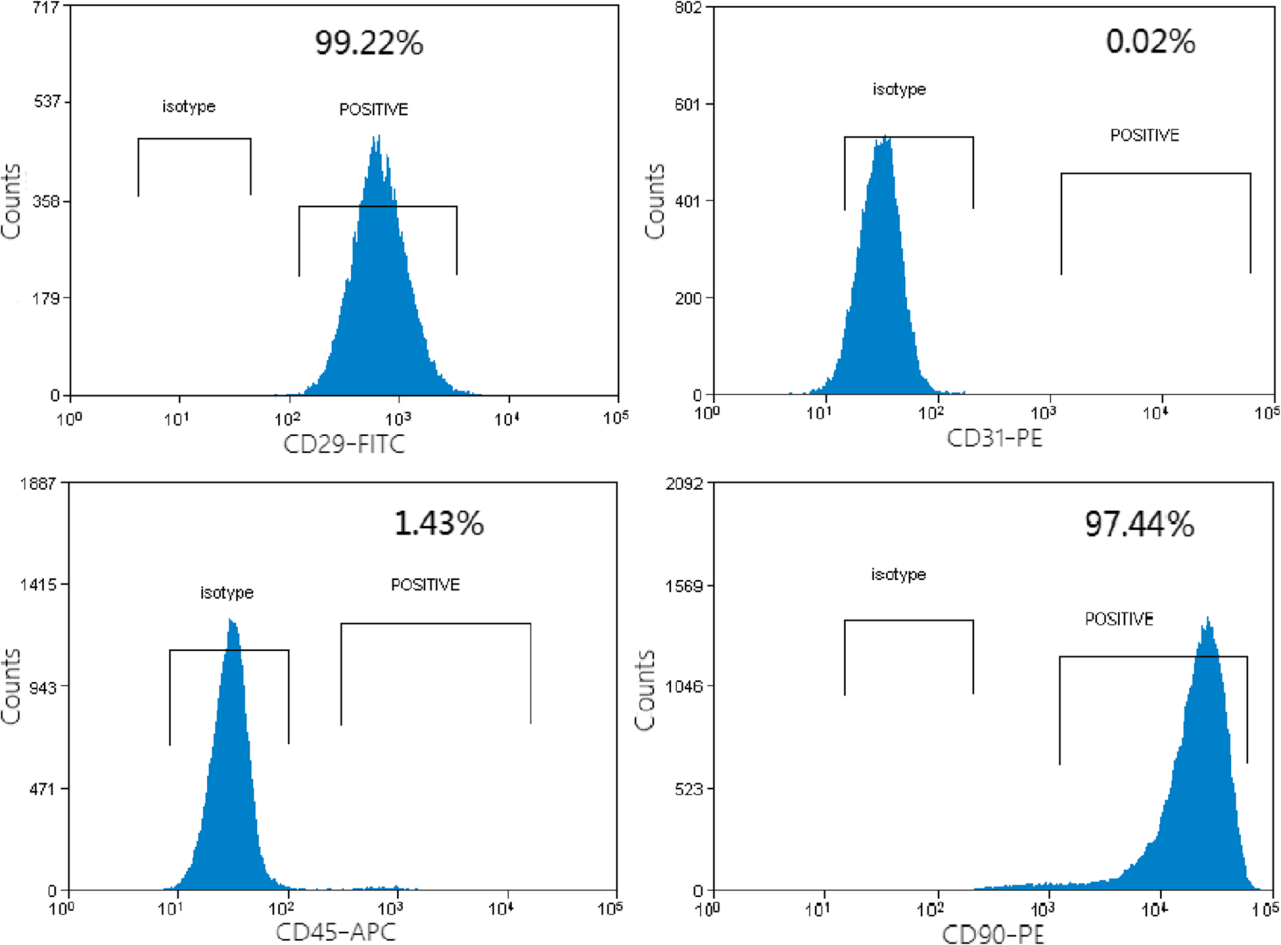
Flow cytometry analysis of cell surface markers in MSCs at passage 3. Fluorescence-activated cell sorting (FACS) analysis for surface antigen profiling of MSC lines. Surface expressions of CD29, CD 31, CD45, and CD90 on MSC lines and control cells were analyzed by using a BD FACS Calibur instrument with BD Cell Quest™ software and off-line analysis by using Flowjo™ software (represented as *blue filling*)

To identify MSCs, phenotypes and multiple differentiation capacities of cultured BM-derived adherent cells at passage 3 were analyzed, respectively. As shown in (Fig. 2), more than 95 % of these cells were positive for CD29, CD90, but negative for CD31, CD45.

In order to confirm whether prepared MSCs could differentiate into two lineages such as osteogenic and adipogenic lineages, we finished the differentiation ability analysis of osteogenic and adipogenic differentiation induction.

**Fig. 3:**
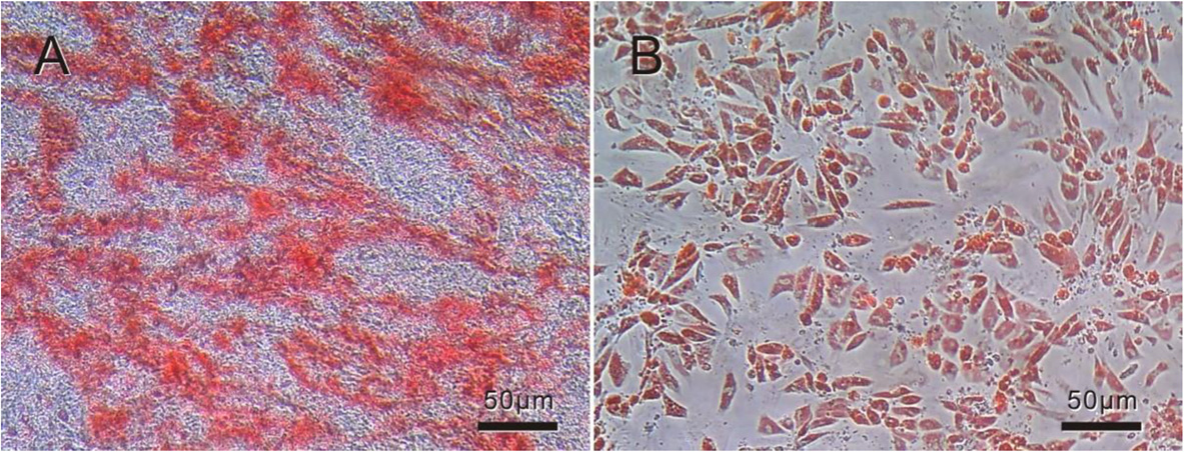
Osteogenic and adipogenic differentiation of MSCs. **a** Calcified colonies after osteogenic induction stained with alizarin red. **b** Lipid droplet accumulated in an adipo-inductive medium stained with Oil Red O

Firstly, osteogenic differentiation was induced in putative MSC lines by culturing cells in an osteo-inductive medium. After 3 weeks in an inductive medium, the treated population contained numerous positive cells widely distributed in the dish and others grouped into colonies. Alizarin red staining of the extra-cellular calcium in differentiated cells, as shown in Fig. 3a, indicated osteogenic differentiation of MSCs into osteoblasts.

Afterward, adipogenic differentiation of putative MSC lines induced by using adipo-inductive media resulted in massive lipid droplet accumulation as demonstrated by positive staining with Oil Red O (Fig. 3b). Lipid droplets were detectable even after 3 days, but a period of 2 weeks was necessary to accomplish maximal lipid accumulation.

In addition, the differentiating abilities of osteogenic and adipogenic lineages of MSCs were assessed in our study as previously described (Fig. 3) [11]. These results indicate that the cultured cells possessed the characteristics of MSCs.

### Preparation and Characterization of CdSe/ZnS Quantum Dots

**Fig. 4:**
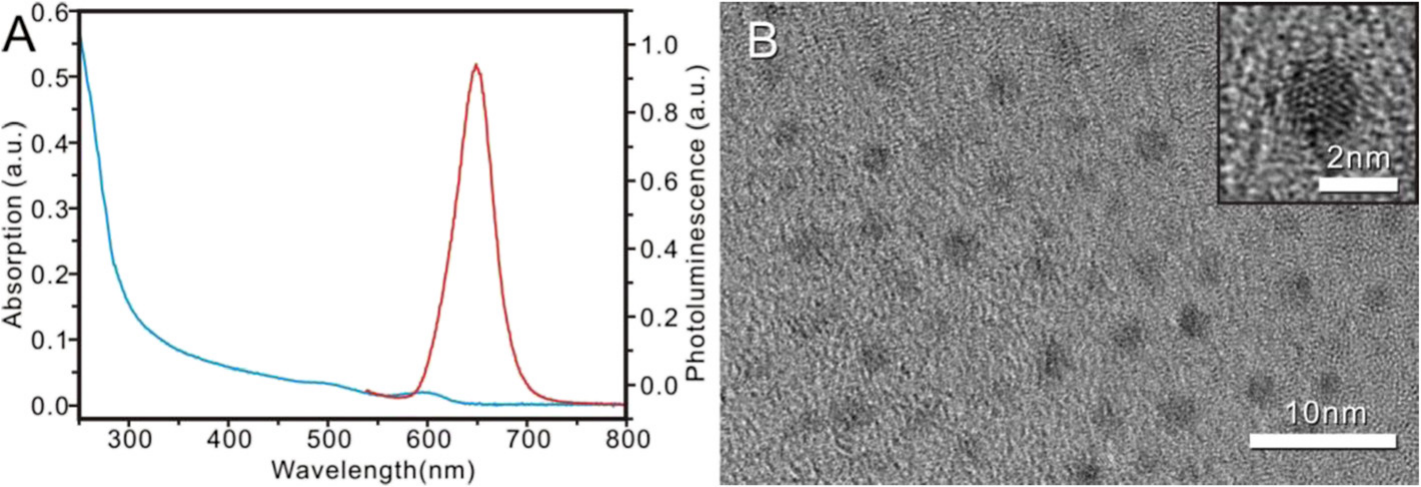
Characteristics of synthesized CdSe/ZnS quantum dots. **a** The absorbance and emission spectra of synthesized QDs. The *blue lines* represent absorbance (*the curves from the upper left to the lower-middle part*) and the *red lines* represent the photoluminescence (*the curves with an obvious protrusive shape*) of the QDs at an emission peak of 655 nm. **b** TEM image of CdSe/ZnS QDs. Insets in **b** showed the HRTEM images of core–shell QDs

As shown in Fig. 4a, synthesized CdSe/ZnS QDs exhibited strong fluorescence signal and narrow emission spectra. Figure 4b shows a typical TEM image of the CdSe/ZnS QDs. The QDs have a narrow size distribution of 4.8 nm in diameter. The existence of lattice planes on the HRTEM confirms the good crystallinity of the CdSe/ZnS core–shell structure. With the ZnS coating, the emission peak of CdSe/ZnS appeared at the wavelength of 655 nm. The shell could not only enhance the core’s anti-oxide ability but also improved its stability and decreased the cytotoxicity.

### Effects of CdSe/ZnS QDs on the Differential Ability of MSCs

**Fig. 5:**
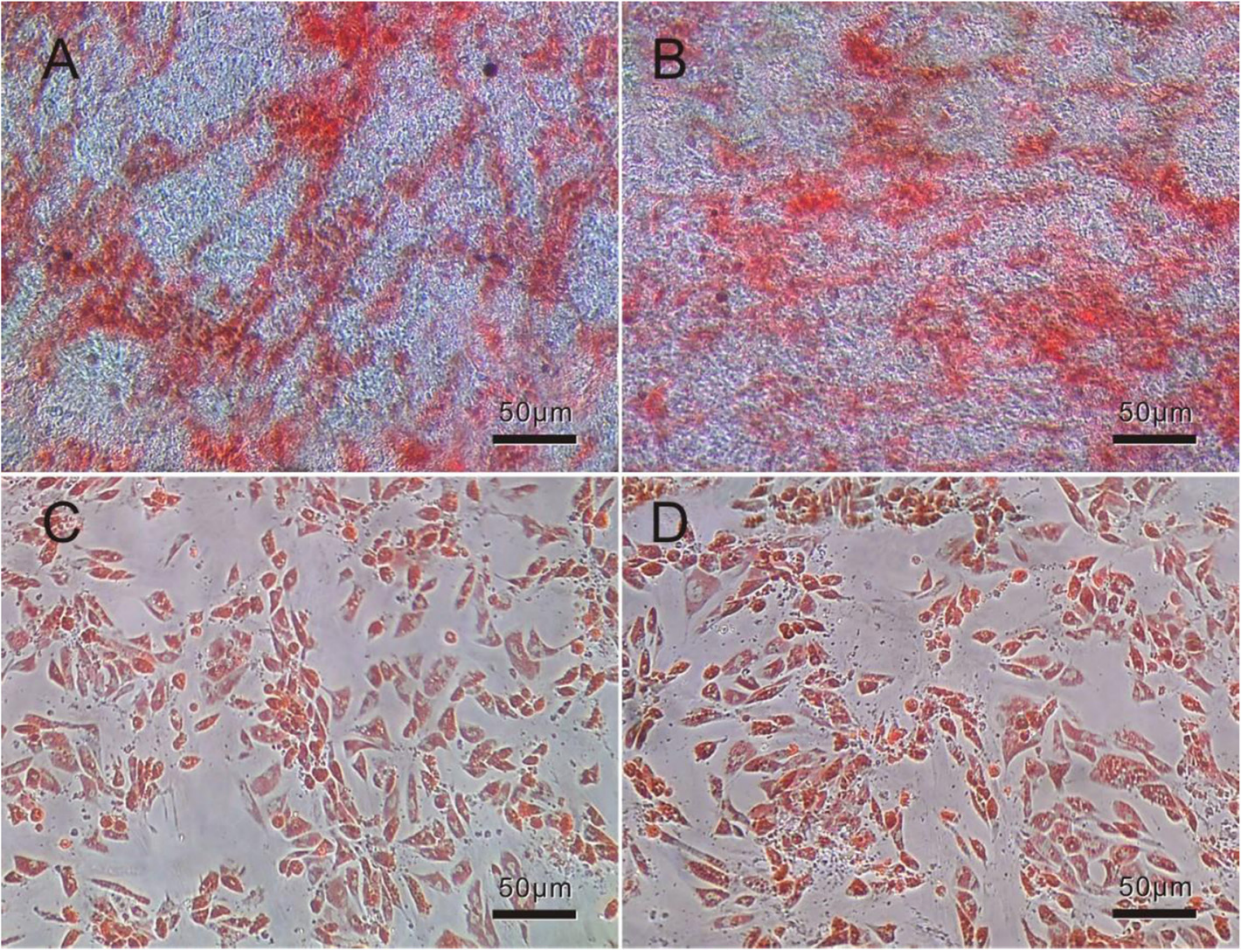
The differentiation ability variation of hMSCs with 16 μg/ml CdSe/ZnS QDs. **a**, **c** The morphology of the osteogenic and adipogenic lineages differentiated from hMSCs under the condition of QDs. **b**, **d**. The morphology of the osteogenic and adipogenic lineages differentiated from hMSCs without QDs

When the MSCs were differentiated under incubation with 16 μg/ml CdSe/ZnS QDs for 2 weeks, the morphology of final cells were compared with the MSCs without QDs. The compared results showed that the CdSe/ZnS QDs had negligible effects on the differentiation ability of MSCs in our experiment (Fig. 5a, b) similar to our previous report [9].

### Effects of CdSe/ZnS Quantum Dots on Viability of MSCs

**Fig. 6:**
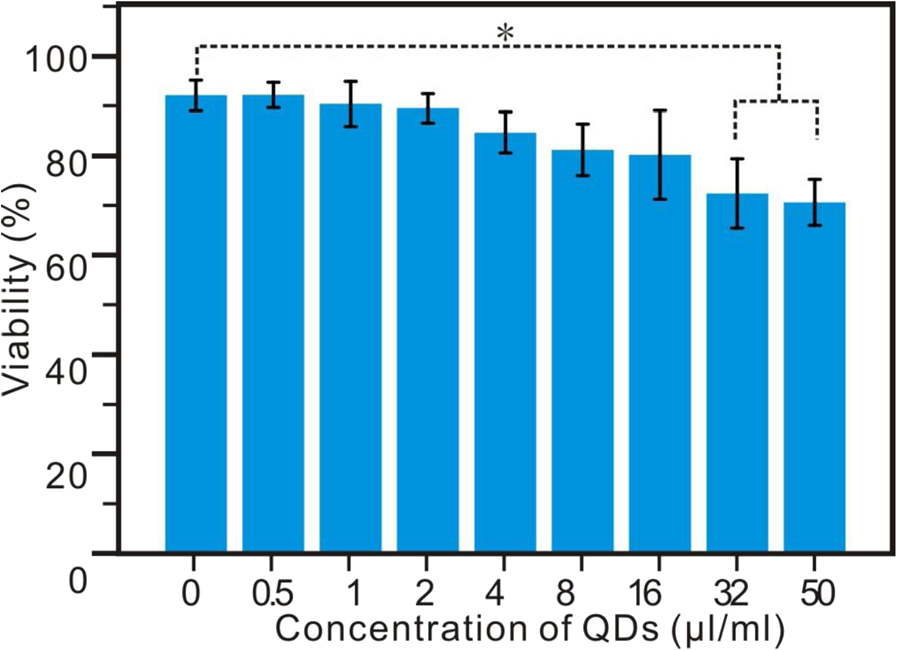
Cell viability assay was observed in MSCs transduced with different concentrations of QDs for 24 h. The survival of MSCs transduced with QDs is compared with non-transduction MSCs. The data, all in triplicate, are shown as the mean ± SD values. *p < 0.05

QDs were transduced into MSCs at various concentrations in a transduction medium for 6 h at 37 °C. Significant cytotoxicity was observed in MSCs transduced with 32 or 50 μl/ml of QDs; however, in addition, no remarkable cytotoxicity was observed, and with less than 16.0 μl/ml of QDs, more than 80 % of the cells were still alive (Fig. 6). Therefore, we chose the QD concentration of less than 16 μl/ml to label the MSCs, so as to study the morphology and fluorescence images with the help of conventional fluorescence microscopy.

### Preparation and Observation of QDs-Labeled MSCs

**Fig. 7:**
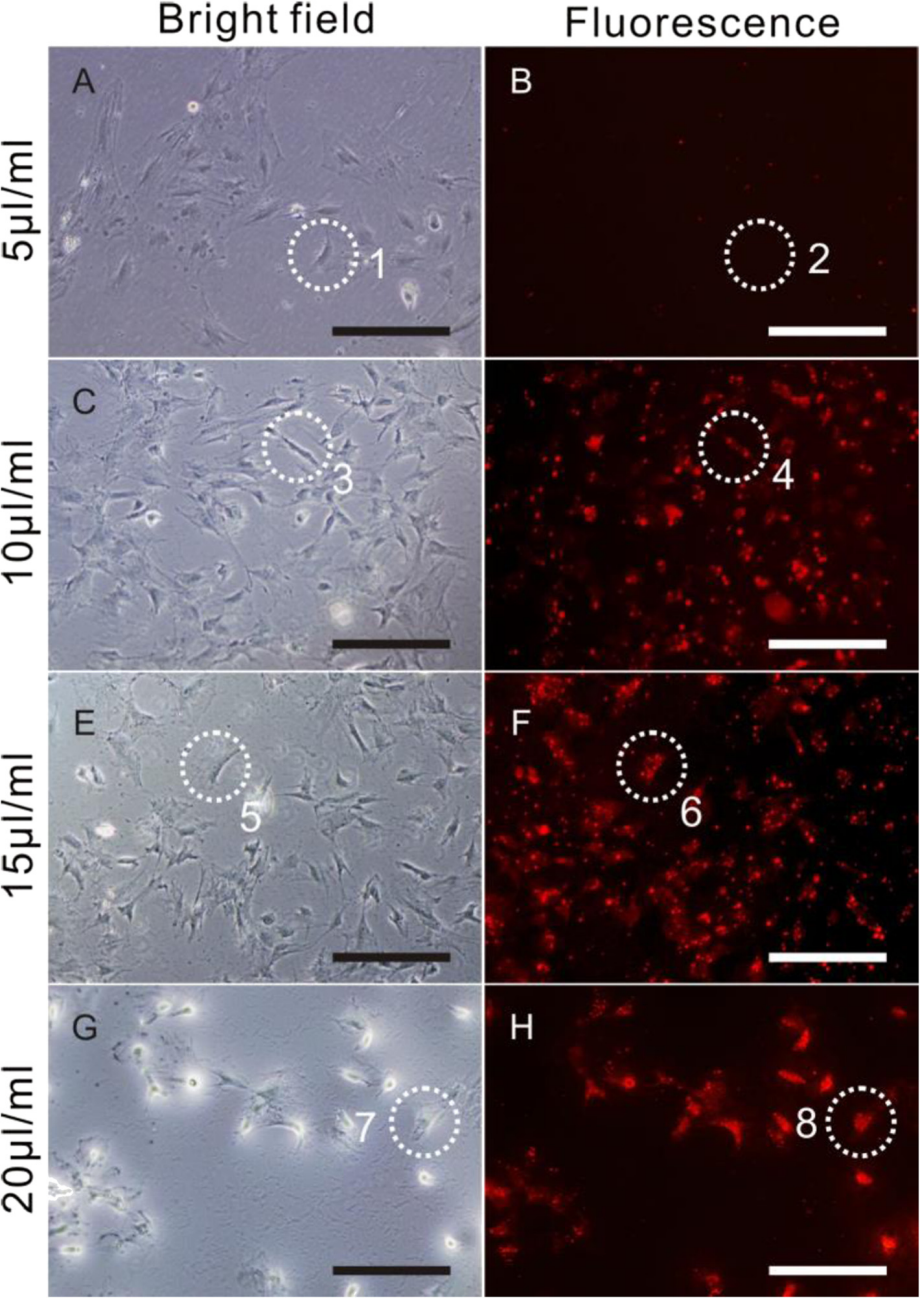
The labeling efficiency of different concentrations of QDs on MSCs. Different amounts of QDs (100 μg/ml) (5, 10, 15, and 20 μl/ml) were incubated with MSCs for 4 h to determine the optimal labeling efficiency. Bright-field images (*left*, **a**, **c**, **e**, **g**) and fluorescence images (*right*, **b**, **d**, **f**, **h**) were taken under inverted fluorescence microscope. *Circle 1* and *circle 2* indicated the same cell image in bright-field and fluorescence background. The remaining *circle 3* and *circle 4*, *circle 5* and *circle 6*, and *circle 7* and *circle 8* were respectively the same single-cell image under bright-field images (*left*) and fluorescence images (*right*)

After incubation with different concentrations of QDs with MSCs for 4 h, the MSC-labeling efficiency was very different, 15 μg/ml QDs exhibited maximal labeling efficiency, and almost all MSCs exhibited strong red fluorescence signals, which showed that almost all MSCs were successfully labeled with QDs. Increasing the concentration beyond 20 μg/ml did not increase the QD internalization, but the ratio of cell death began to rise (Fig. 7). This result suggested that the optimal concentration ratio of QDs was 15 μg/ml.

**Fig. 8:**
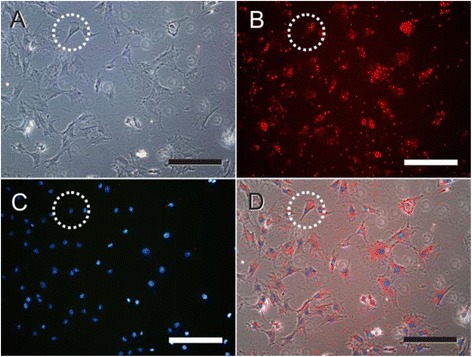
Observation of QDs entering into MSCs. **a** Bright-field images of MSCs treated with QDs. **b** Fluorescence image of cytoplasm labeled by QDs. **c** nucleus stained by DAPI. **d** The overlaid images was merged with ImageJ. *Blue* represents DAPI stained nucleus, and *red* represents QDs. The *four circles* indicated the identical cells. Scale bars are 200 μm

In order to investigate the mechanism of QD-labeling MSC cells, as shown in Fig. 8, QDs were endocytosed into MSCs, and located in the cytoplasm around nucleus, QDs exhibited a strong red color (Fig. 8b), and cell nucleuses exhibited a blue color (Fig. 8c). The overlaid image (Fig. 8d) showed that most of the quantum dots were located inside the cytoplasm. With time elapsed, there were no QDs exocytosised by MSCs, and QDs coexisted with MSCs closely.

### The Measurement of Blood Glucose Levels

**Fig. 9:**
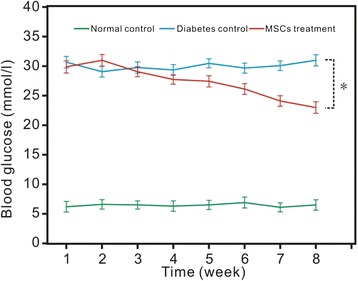
Blood glucose of the three groups for 8 weeks after MSC treatments. We set the time when MSCs were injected as the start of the study. The data, all in triplicate, are shown as the mean ± SD values. *p < 0.05

To verify the treatment of mesenchymal stem cells, we investigated the level of blood glucose after MSC injection (10 rats of each group, including the normal control, diabetes control, and treatment groups). As shown in Fig. 9, FPG of rats in the normal control group maintained within the normal range. FPG of rats in the diabetic control group and MSC treatment group changed significantly. FPG in the diabetic control group and MSC treatment group was higher than the normal control group and diabetic control group. The level of FPG in the diabetic control group was significantly higher than in the MSC treatment group (*p* < 0.05, 8 weeks after MSC transplantation) (Fig. 9).

### In Vivo Imaging and Distribution of QDs-Labeled MSCs

**Fig. 10:**
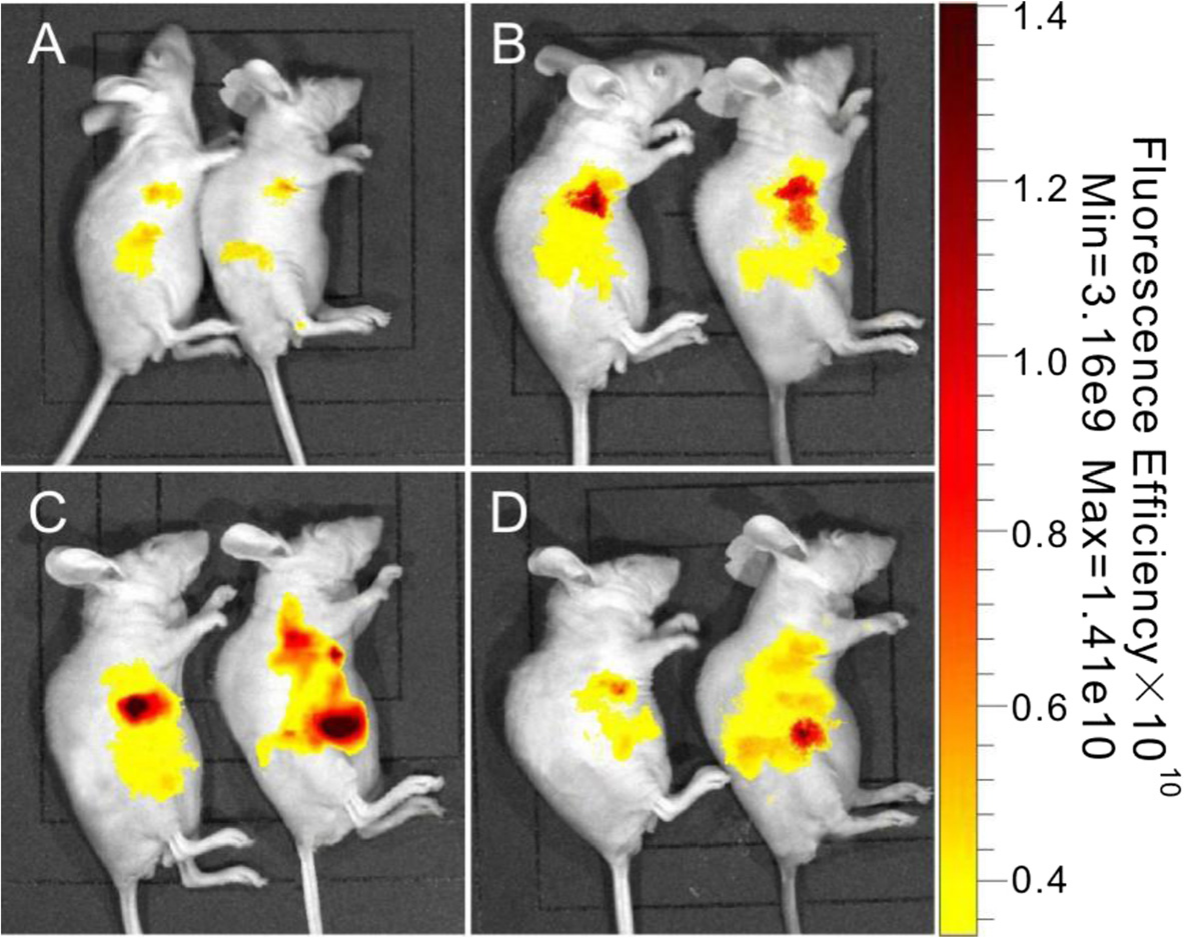
In vivo fluorescence imaging in different time points after tail vein injection of CdSe/ZnS QDs-labeled MSCs. **a**–**d** The time points of 1, 3, 6, and 12 h, respectively

After tail intravenous injection of CdSe/ZnS QDs-labeled MSCs in the diabetes group and normal control group, the fluorescence signals were observed in different time points (1, 3, 6, and 12 h). As shown in Fig. 10a, after CdSe/ZnS QDs-labeled MSCs were injected into mice for 1 h, MSCs accumulated in the lungs and abdominal cavity, and the fluorescence signal strength had no significant difference between the normal control group and diabetes group. After 3 h of injection, significant MSC accumulation in the lungs can be observed in both groups (Fig. 10b). Six hours later, the CdSe/ZnS QDs-labeled MSCs were homed into the pathological tissue of the pancreas and the strongest fluorescence intensity can be captured. In the normal control group, the MSCs were mainly accumulated in the liver. While in diabetic mice, the dominant signals were in the pancreas (Fig. 10c). After injection for 12 h, the signals of QDs-labeled MSCs still can be observed in the diabetes group, but the signal intensity decreased obviously (Fig. 10d).

### Biodistribution of QDs-Labeled MSCs

**Fig. 11:**
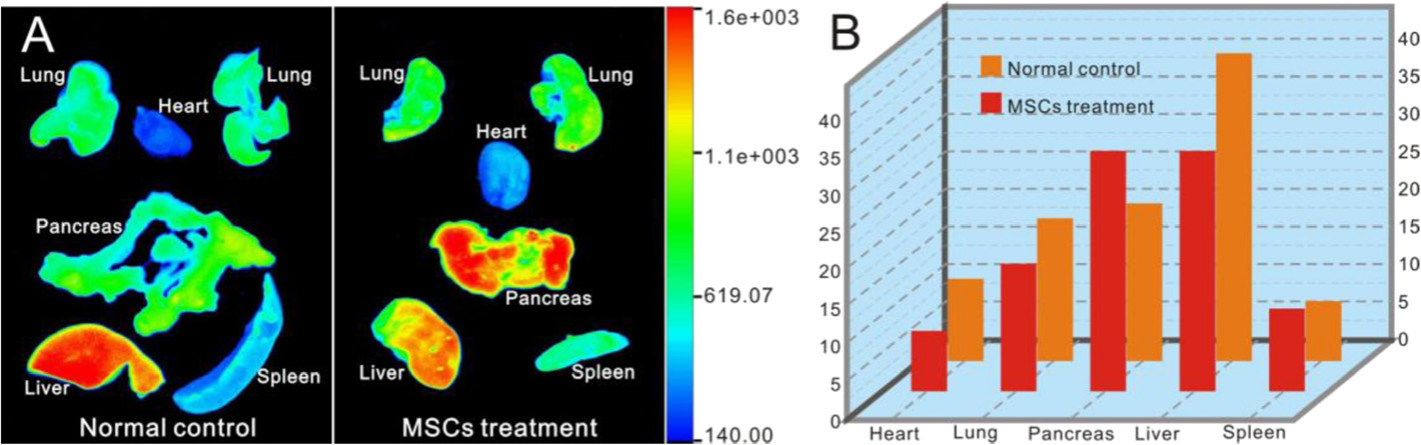
Fluorescence imaging of rats with diabetes transplanted with QDs-labeled MSCs. **a** The ex vivo fluorescence images of five important organs of rats with diabetes. **b** The ratio of the fluorescence intensity (RFI) of each organ equals the fluorescence intensity of the organ divided by the total fluorescence intensity of all five organs (heart, lungs, pancreas, spleen, and liver) in the normal control group and MSC treatment group (multiplied by 100)

As shown in Fig. 11a, five important organs (heart, lungs, pancreas, spleen, and liver) were harvested and imaged in the Bruker In-Vivo F PRO system. The fluorescence images exhibited different intensities of fluorescence signals within and between the control and experimental groups. The pancreas fluorescence signal of diabetic rats was much higher than that of the control groups. Meanwhile, in the control group, the strongest signal of QDs was mainly accumulated in the liver. This result demonstrated that the QDs-labeled MSCs could target and homing to the pathological pancreas initiatively, play the important role in the treatment of diabetes.

After 6 h of MSC transplantation, in order to analyze the accumulation ratio of QDs, the ratios of fluorescence intensity (RFI) analysis was performed in five organs (Fig. 11b). The RFI results showed that the RFI of MSCs in the pancreas of diabetic rats was about 32 %, while that in the normal control group rats was about 18 % (*p* < 0.05, triplicate of each groups). However, the RFI of the liver in the normal control group was much higher than that of the diabetes group (*p* < 0.05, triplicate of each group). These data suggest that in vivo MSCs can migrate and enter into the pancreas tissues actively, can be introduced for pathological imaging of the pancreas tissue, and deduce the therapeutic function initiatively.

### Potential Mechanism

Previous studies have revealed that MSCs are capable of reducing glucose levels in animals or subjects with type 1 diabetes [12]. The underlying mechanism of the therapeutic effect of MSCs on hyperglycemia might involve islet regeneration [13]. Several studies highly indicate that the unique immunomodulatory effects of MSCs seemed to be closely associated with decreasing hyperglycemia. MSCs also exerted anti-inflammatory effects that might be important in maintaining peripheral tolerance [14, 15].

In our study, the blood glucose of the normal control group and MSC treatment group changed significantly at 2 months after MSC injection. The FPG of the MSC treatment group dropped more than 5 mmol/l, while the diabetic control group and normal control group make no difference at the beginning of the study, and this phenemenon was similar to previous reports [16, 17].

In order to clarify the potential mechanism, we prepared QDs-labeled MSCs and observed their in vivo distribution. QDs own obvious advantages compared with traditional fluorescent dye molecules [18]. Specifically, QDs have a broad absorption range from ultraviolet (UV) to visible light, but exhibit distinct narrow emission spectrum, resistant to photochemical degradation; therefore, QDs are very suitable for tracking MSCs and monitoring cell biological changes in vivo [19–21].

We selected SD rats to prepare a diabetes model [22–24]. With the help of the Bruker In-Vivo F PRO system, the transplanted MSCs were observed to accumulate in the liver in the normal control animals, few MSCs in the pancreas tissues, and the RFI analysis showed that the accumulation rate of MSCs in the liver was about 41 %. While in diabetic rats, the accumulation rate of the transplanted MSCs in the liver was decreased to about 32 %, and the accumulation rate in the pancreas increased to about 32 %. Accordingly, distribution difference between the two groups should be closely associated with pancreas injury, which also confirms that MSCs can target and accumulate to the injured organ [10, 25–30]. Our results also indirectly suggest that quantum dots do not alter the self-replication and differentiation potential of MSCs [7, 31, 32] and exhibit great potential in tracking MSCs in vivo [33].

Regarding the molecular mechanism of MSC homing, the transplanted MSCs were observed to accumulate in the liver in normal control, which may be caused by Kupffer cells with strong phagocytosis in hepatic sinusoid [31, 34]. The accumulation of transplanted MSCs in the pancreas of diabetic rats may be closely associated with chemokines [35, 36]. Our previous studies confirmed that CCL19/CCR7 and CXCL12/CXCR4 axis loops played key roles in the targeting of MSCs to in vivo gastric cancer [37–39]. Therefore, we predict that CCL19/CCR7 and CXCL12/CXCR4 axis loops also may play key roles in the targeting of MSCs to in vivo injured pancreas. The concrete mechanism is under way.

## Conclusions

We investigated the behavior and organ-specific accumulation of transplanted MSCs labeled with QDs in a rat model of type 1 diabetes. Using the Bruker In-Vivo F PRO system, the accumulation rate of MSCs in the pancreas of rats in the diabetes group was increased compared with the normal control group. The MSC-injected diabetic rat group displayed lower blood glucose levels. Our results highly indicate that QDs-labeled MSCs can target in vivo pancreatic tissues of diabetic rats, and decrease the blood glucose levels, and then, in vivo transplanted MSCs may own clinical therapeutic prospect to diabetic patients.
